# How to Differentiate Pronator Syndrome from Carpal Tunnel Syndrome: A Comprehensive Clinical Comparison

**DOI:** 10.3390/diagnostics12102433

**Published:** 2022-10-08

**Authors:** Adrian Andrzej Balcerzak, Kacper Ruzik, Richard Shane Tubbs, Marko Konschake, Michał Podgórski, Andrzej Borowski, Marek Drobniewski, Łukasz Olewnik

**Affiliations:** 1 Department of Anatomical Dissection and Donation, Medical University of Lodz, 90-419 Lodz, Poland; 2 Department of Neurosurgery, Tulane University School of Medicine, New Orleans, LA 70112, USA; 3 Department of Neurosurgery and Ochsner Neuroscience Institute, Ochsner Health System, New Orleans, LA 70121, USA; 4 Department of Anatomical Sciences, St. George’s University, St. George FZ818, Grenada; 5 Department of Surgery, Tulane University School of Medicine, New Orleans, LA 70112, USA; 6 Department of Structural and Cellular Biology, Tulane University School of Medicine, New Orleans, LA 70112, USA; 7 Department of Neurology, Tulane University School of Medicine, New Orleans, LA 70112, USA; 8 Institute of Clinical and Functional Anatomy, Medical University of Innsbruck, Innsbruck, TIR 6020, Austria; 9 Department of Diagnostic Imaging and Interventional Radiology, Medical University of Lodz, 90-419 Lodz, Poland; 10 Orthopaedics and Pediatric Orthopaedics Department, Medical University of Lodz, 90-419 Lodz, Poland

**Keywords:** carpal tunnel syndrome, pronator syndrome, median nerve, differential diagnosis

## Abstract

The diagnostic process that allows pronator syndrome to be differentiated reliably from carpal tunnel syndrome remains a challenge for clinicians, as evidenced by the most common cause of pronator syndrome misdiagnosis: carpal tunnel syndrome. Pronator syndrome can be caused by compression of the median nerve as it passes through the anatomical structures of the forearm, while carpal tunnel syndrome refers to one particular topographic area within which compression occurs, the carpal tunnel. The present narrative review is a complex clinical comparison of the two syndromes with their anatomical backgrounds involving topographical relationships, morphology, clinical picture, differential diagnosis, and therapeutic options. It discusses the most frequently used diagnostic techniques and their correct interpretations. Its main goal is to provide an up-to-date picture of the current understanding of the disease processes and their etiologies, to establish an appropriate diagnosis, and introduce relevant treatment benefiting the patient.

## 1. Introduction

Both pronator syndrome (PS) and carpal tunnel syndrome (CTS) are pathologies associated with entrapment of the median nerve (MN) [1,2,3,4]. The main difference between their etiologies lies in the location of compression: PS can be caused by compression of the MN as it passes through the anatomical structures of the forearm; CTS is caused by compression at the carpal tunnel. Both conditions can be influenced by the anatomical topography of the muscles, their anatomical variability, their associated fasciae, and the positions of their ligaments. They are also influenced by environmental factors, such as functional overload of the muscle fibers. In addition, microinjuries and muscle tears also cause inflammation and swelling and thus reduce space, increasing carpal tunnel pressure (in CTS) and compressing the nerve [1,3,5,6,7].

However, the MN can also be compressed in other pathological processes, not directly related to muscle fiber injury or inflammation, such as tumors [8] or iatrogenic [9] injuries to the MN, resulting in symptoms typically encountered in PS or CTS.

PS is a rare compressive neuropathy associated with an insidious onset of indistinct pain in the proximal forearm, paresthesia in the MN distribution and pain with activity, caused by compression of the MN in the proximal forearm by various anatomical structures [10,11,12,13,14,15]. In contrast, CTS comprises a group of symptoms related to MN compression at the wrist caused by increased carpal tunnel pressure [3,16,17]. The diagnosis and differentiation of these syndromes remain a challenge for clinicians, as the scientific literature provides little to no reliable information on precise diagnosis or treatment algorithms; as such, it is difficult to establish a diagnosis and decide on effective treatment [18,19,20,21,22].

The aim of the present study is to present a comprehensive clinical comparison of PS and CTS based on their morphologies. It also discusses diagnostic and differentiation processes, summarizes various treatment options along with their effectiveness, and provides clinicians with an overview of the current state of knowledge in this area.

## 2. Methodology

A literature review was performed of the anatomy, etiology, and clinical aspects of carpal tunnel syndrome (CTS), and pronator syndrome (PS). An electronic search was conducted in four databases: (1) The National Library of Medicine (MEDLINE/PubMed) via Ovid; (2) Google Scholar; (3) SCOPUS; and (4) Cochrane Central Register of Controlled Trials (CENTRAL) up to March 2022. The types of the studies that met the inclusion criteria were:

- Systematic studies related to carpal tunnel syndrome and/or pronator syndrome anatomy, etiology, diagnosis, and/or treatment.
- Case reports related to carpal tunnel syndrome or pronator syndrome anatomy, etiology, diagnosis, and/or treatment.
- Reviews related to carpal tunnel syndrome or pronator syndrome anatomy, etiology, diagnosis, and/or treatment.
- Book chapters related to carpal tunnel syndrome or pronator syndrome anatomy, etiology, diagnosis, and/or treatment.

The searches comprised the following keywords and their combinations: pronator teres syndrome, carpal tunnel syndrome, comparison, compression, median nerve, symptoms, anatomy, morphology, etiology, treatment, surgery, differential diagnosis, and clinical aspects.

Data from over 120 relevant articles were gathered, analyzed, and included in this comprehensive review.

## 3. Median Nerve Course and Its Variations

Both pronator [2] and carpal tunnel [23] syndromes are caused by compression of the MN, which originates from C5 to C8 and T1 at the brachial plexus level [24], accompanies the brachial artery initially on the lateral side, crosses it at mid-arm to the medial side, and continues its course distally between the biceps brachii and brachialis muscles [25,26]. After reaching the antecubital fossa, it runs under the lacertus fibrosus (LF), a fibrous structure originating from the ulnar border of the biceps brachii [27]. Its course after reaching the forearm can vary depending on the presence of anatomical variations in individual muscles, which can modify its course and potentially create predisposing pressure points [15].

The nerve most commonly travels between the ulnar and humeral heads of the PT [28,29,30,31,32], but Olewnik et al. [15] observed a variation in this section of MN, in which it passes under both heads or deeply under only one (humeral) head. Wertsch et al. [1] observed that the anterior interosseous branch of the MN is particularly subject to compromise near its origin.

Distally to the PT, the nerve descends deep to the two heads of the flexor digitorum superficialis (FDS) and continues distally into the forearm between the flexor digitorum profundus (FDP) and FDS [12,33,34]. Near the retinaculum flexorum, it descends lateral to the musculus FDS and passes to the palm between the muscle and the retinaculum flexorum. In the forearm and palm, it supplies motor branches to the flexors and sensitive branches to the skin of the lateral side of the palm [35,36]. Over the years, some morphological variations in the course of MN have been reported [35], but normal morphology was observed in 82.6% of 1000 cases [35].

## 4. Etiology of Pronator Syndrome

By definition, pronator syndrome is a compressive neuropathy of the median nerve in the proximal forearm [13]. The morphology of PS depends on the course of the MN and the topographic structures surrounding it [12]. As compression can potentially be caused by many structures in the forearm, an overview of the course of the MN and its topographical relationships is necessary.

The pronator teres muscle (PT), from which the syndrome takes its name, has been repeatedly observed to be the most common cause of nerve compression and induction of symptoms [4,18,20,37]. However, researchers have described other structures that can provoke PS symptoms, such as the lacertus fibrosus (LF), the flexor digitorum superficialis fibrous arch (FDS), ligaments of the supracondylar process, and the accessory head of the flexor pollicis longus (FPL) (Gantzer muscle) [4,18,20,37,38]. The percentages of causes of PS vary across studies, ranging from 33% to 76% when the etiology is connected to the PT, 0% to 42% to the LF, and 14% to 36% to the FDS [4,18,20,37]. In addition, 1–2% of the population possess a ligament of Struthers, a fibrous structure extending from the distal medial humeral diaphysis to the medial epicondyle, which can function as a PT origin and, in rare cases, as a site of MN compression [24]. Other rare sites of nerve compression include the accessory head of the flexor pollicis longus (Gantzer muscle), the net of blood vessels crossing the MN at PT level, the exostosis of the radius, adherent fascial bands of the brachialis, the brachialis itself, and also the ulnar part of the flexor carpi ulnaris [1,12,19,38,39,40].

### 4.1. Pronator Teres Muscle

The PT muscle forms the superficial layer of muscles of the anterior forearm group along with the flexor carpi ulnaris, palmaris longus and flexor carpi radialis [33,34,36]. This fusiform muscle most commonly consists of two heads: the humeral (superficial) head, attached proximally on the medial intermuscular septum of the arm and to the medial epicondyle of the humerus, and the ulnar (deep) head, which originates from the coronoid process [7,15,41]. Both heads merge in their distal course and together form a common flexor tendon, which inserts on the middle part of the lateral side of the radius [33,34,36]. However, up to 45% of the population have a variation in which the ulnar head of the PT is absent and is replaced by a distinct fibrous structure [13,20]. In adults, the PT can be palpated around 6 cm distal to the elbow crease and 4 cm lateral to the medial epicondyle [7,11,12].

The PT is typically supplied with blood by the radial artery, which often runs anteriorly to its tendon [15]. However, if its origin is low, its course can be observed beneath the PT [33]. The ulnar artery also can pass through the PT muscle, providing it with a vascular net [42].

Innervation is provided by the median nerve (MN). If both heads of the PT are present, three types of innervation can be observed, as classified by Olewnik et al. [15]. Type I—one muscular branch arises from the main trunk of the MN; Type II (most common)—two muscular branches arise from the main trunk of the MN; and Type III—three muscular branches arise from the main trunk of the MN. When the ulnar head was absent, two muscular branches were observed [15].

The course of the MN and its topographical relationship to the PT muscle play key roles in compression symptoms [37,41,43]. Therefore, morphological variations within this muscle could be directly connected to the cause of MN compression in this area [7,15,20]. Dellon and Mackinnon [44] report that when the origin of the humeral head was at least 2 cm proximal to the medial epicondyle, the fascia connected to the LF was in a position to compress the MN. Werner et al. [43] showed that in 78% of cases, the nerve penetrated the superficial head of the PT, and additional bands of muscle itself compressed the MN.

Other commonly observed sources of MN compression include thickened intramuscular tendinous and fibrous bands that arise from both PT heads [7,20,41]. Unfortunately, existing evidence is not sufficient to clearly identify which structure is most often observed during surgery [12].

### 4.2. Lacertus Fibrosus

Additionally, named the bicipital aponeurosis in the literature [12,45,46], the LF is a fascial extension that extends from the distal part of the ulnar aspect of the biceps tendon, crosses the antecubital fossa obliquely, travels anteriorly to the MN, and merges with the deep fascia of the flexor section of the forearm muscles [7]. Passive pronation of the biceps tendon drags on the structure, compressing those lying inferiorly, including the MN, which can cause PS symptoms [37]. According to Dellon et al. [44], this site of nerve compression is especially common when it coincides with a high origin of the humeral head of the PT. Another important, though very rare, variation described by Kumar [47] occurs when the MN pierces the LF along with the brachial artery, which can be important for diagnosing pathologies in this area and operating on them.

Caetano et al. [45] found the LF to be thickened in 70% (42/60) of sixty upper limbs. In this subgroup, the LF lay directly on the MN in 64.3% of cases (27/40), and a high insertion of the humeral head of the PT in 40.5% (17/42), which was interposed between the LF and MN. According to the authors, these results indicate that a thickened LF can narrow the space through which the MN passes and thus being a potential factor in nerve compression.

Seitz et al. [48] describe seven cases of acute MN compression in the antecubital fossa which resulted from elbow injuries. During physical examination, elbow flexion against a counterforce resulted in severe pain radiating to the lower forearm. Surgical decompression revealed partial rupture of the myotendinous junction of the biceps brachii, creating increased tension across the MN by a tethered LF. Surgical decompression resulted in complete relief of symptoms in all seven cases.

Patients who report symptoms affecting only one limb may also be suspected of bilateral predisposition to MN compression by the LF. This was confirmed by Ishikhov et al. [22], who found that bilateral LF entrapment of the MN at the elbow is possible.

### 4.3. Flexor Digitorum Superficialis (FDS)

Formerly known as the flexor digitorum sublimis [49], this is the largest extrinsic flexor of the forearm. It forms the intermediate muscle layer between the superficial and deep muscle groups of the forearm [50]. The FDS is the primary flexor of the proximal interphalangeal joints of the middle phalanges, but it also assists in flexion of the metacarpophalangeal (MCP) joints [51]. It most commonly has a humeroulnar head, which originates from the medial epicondyle, and either the interosseous membrane or the most radial surface of the ulna and radial heads (which is a much wider origin and arises obliquely from the volar radius) [12,44].

It has been observed [41,44,52] that the two heads can jointly or severally form a tendinous or fibrous aponeurotic arch under which the MN travels before continuing distally. Five types of anatomical variation of the FDS muscle and tendon were described by Elias et al. [53]: Type I—muscle attached to FDS tendon proximally and index finger distally; Type II—muscle originating from flexor retinaculum and attached to FDS tendon in distal palm; Type III—additional digastric muscle inserted on base of middle phalanx of middle finger, passing under carpal tunnel; Type IV—muscle belly corresponding to index finger and extending into carpal tunnel; Type V—muscle crossing the MN anteriorly, attached to the FDS of the middle finger by a tendon and the deep surface of the transverse carpus ligament. Elias et al. [53] emphasize that Types I and IV can cause intermittent symptoms related to MN compression.

To summarize, the FDS can play a role in PS by forming a tendinous or fibrous arch by its heads [41,44,52] and in CTS due to the specific muscle and tendon morphology in its distal course [51,53].

### 4.4. Other Anatomical Structures Involved in PS

Aside from the muscles most commonly involved in PS (the PT, LF and FDS), there are cases in which authors have described much rarer topographical structures predisposing to MN compression, such as the ligament of Struthers [13,54], Gantzer’s muscle [44,55,56], vascular arcades crossing the MN, exostosis of the radius, the fibrous component of the FCR, or the fascial bands of the brachialis muscle [1,19,39].

The ligament of Struthers is a fibrous structure extending from the distal medial diaphysis to the medial epicondyle [12] observed in 1–2% of the population. It can also form the supracondylar process, an anteromedial bony projection of the humeral diaphysis [24,57], and can be an accessory origin for the PT belly compressing the MN [7]. Direct MN compression by the ligament of Struthers is observed only in individual cases [13,54].

The Gantzer muscle is an accessory head of the flexor pollicis longus, commonly arising from the medial epicondyle, coronoid process or interosseous membrane connecting the FDS and the flexor digitalis profundus [44,55,56]. Its incidence varies among studies from 20% to 75% of limbs, but although it has been suggested as a possible site of compression, this has been observed only a few times [12].

PS is more common in women, and around the fourth and fifth decades of life, with no evidence supporting a correlation with hand dominance [4,20,37]. Although it is considered a chronic entrapment neuropathy, patients occasionally present with acute muscle sprains or episodes that precipitate a PS [37,48].

## 5. Etiology of Carpal Tunnel Syndrome

Carpal Tunnel Syndrome most commonly is described as group of symptoms related to MN compression in the carpal tunnel: a passageway on the palmar side of the wrist which connects the forearm to the hand [3]. It is limited by carpal bones—hamate, capitate, trapezoid, trapezium—and a fibrous band arching from pisiform bone and hook of the hamate bone to the tubercle of scaphoid bone and the medial part of palmar surface of trapezium bone—flexor retinaculum [33]. CTS can be divided into an acute form, often caused by a rapid and sustained rise of pressure in the carpal tunnel, and a chronic form, which is associated with pathological processes over a longer period of time [23]. Although Paget [58] reports that the acute form of CTS most commonly results from a radius fracture, it can also be associated with burns, coagulopathy, local infection, and injections, all relating directly or indirectly to an increase in carpal tunnel pressure [23].

The chronic form of CTS remains an interesting area of study due to its low incidence of identified causes. Aroori and Spence [23] state that only 50% of cases can be related to a definite pathological mechanism and that these are classified into three groups: local causes, regional causes, and systematic causes (Table 1).

Osiak et al. [60] point out that a persistent median artery is an important factor in CTS etiology because it can compress the MN; however, it occurs in only 2.8% of cases [61].

Most importantly, CTS accounts for up to 62% of reported occupational diseases [62]; however, the literature is unclear as to whether it has a higher incidence in workers who are involved in repetitive work involving high forces [63,64,65,66,67,68]. Moreover, the odds ratios for repetition, force and their relationship also vary in studies describing the link between CTS and occupation [23,63,64]. This inconsistency could be related to the wide variety of criteria used to diagnose CTS, but even since more rigorous methods to establish diagnosis were introduced during the 1990s, both positive and negative links between occupational factors and CTS have been reported [69,70,71,72]. It has been proposed that the etiology of CTS involves a combination of several factors in addition to occupational risk [23]. Various studies indicate that it is correlated with a high body mass index [73,74,75,76] and unhealthy habits and lifestyle [76,77], including alcohol, tobacco, and caffeine consumption [78].

The pathogenesis of CTS is not clear [23,60]. It is hypothesized that mechanical compression resulting from increased interstitial pressure within the carpal tunnel causes local ischemia and therefore demyelination of MN fibers; sensory fibers are usually affected before motor fibers, translating into the clinical picture and onset symptoms [60].

A recent study by Zyluk et al. [79] reviewed the role of genetic factors in the etiology of CTS, describing a genetic predisposition based on pathomechanisms that involve the synthesis and breakdown of collagens (related to the COL1A1, COL5A1, and COL11A1 genes) and proteins that can protect against oxidative stress. Genetic predisposition can be suspected when a young patient presents early onset of symptoms, CTS occurs repeatedly in the same family, or when the incidence of CTS in relatives is greater than that in the general population. A genetic predisposition can be also suspected when the CTS symptoms are bilateral [80].

## 6. Symptoms and Differential Diagnosis

Physical examination is still considered the gold standard for diagnosis of both CTS [81] and PS [12].

Patients with PS most commonly describe symptoms as aching in the proximal forearm with paresthesias in areas innervated by the MN [15,18,48]. Persistent elbow flexion and forearm supination, forearm pronation and repetitive grasping can stretch the PT and lacertus, pushing them against the MN and thus producing symptoms [19]. Some studies have shown that patients sometimes report numbness in the volar radial three and a half fingers and the thenar eminence [37,48].

CTS patients report tingling, numbness, and pain within the MN distribution, particularly the thumb and the index and middle fingers, worsening at night. The pain often radiates proximally to the forearm or shoulder [82].

During physical examination, patients with PS present tenderness with palpation in the area of the proximal forearm. Indentation of the flexor-pronator mass that can be palpated below the medial epicondyle can suggest that the compression is caused by the LF [54]. CTS patients sometimes present weakness and atrophy of the thenar muscles associated with sensory loss in the affected fingers [82].

The incidences of CTS and PS differ significantly. PS is diagnosed in only 1.1% of compressive neuropathies of the upper extremity [57]. However, CTS has been found to be the most common misdiagnosis for PS, constituting 32% [19] and 49% of cases [18], where the patient had undergone unsuccessful CTS decompression before presentation.

It has been proposed that the motor strengths of all the muscles innervated by the compressed nerve, particularly the FPL, FDP, and flexor carpi radialis, should be evaluated individually and compared with those of the other upper extremity [12]. Most authors describe only minimal objective motor deficits upon examination, which could be attributed to the sensory fibers being injured before the motor fibers [60]. However, the literature also provides studies hypothesizing that muscle weakness is easily missed in traditional examination and is actually common in PS [11,19,48,83].

During physical examination, a diagnosis of PS can be established using mainly static examinations, the pronator compression test, and also certain movements of the forearm related to muscle positions provoking symptoms as a dynamic test.

Applying sustained pressure for 30 s over the PT in places where the MN courses can provoke paresthesias, which constitute a positive test result [7,11,18,57]. A positive compression test is one of the most common findings in surgically treated PS patients [37,43]. No studies indicate that a PT pressure test is positive in cases of isolated CTS.

Dynamic examination of muscles of the forearm can navigate clinicians during the diagnostic process, enabling subjective tests to be conducted on the PT, LF and FDS as potential sites of MN compression [7,12]. However, these should be performed with great accuracy owing to the strict range of muscle activities when the movements are performed.

To test the PT specifically as a compression site, the patient should pronate the forearm against resistance by the examiner’s hand with the elbow joint flexed from 0 to 45 degrees. Increased pain sensation or paresthesia can indicate MN compression against the PT [7,20,39,57].

The LF should be tested by having the patient flex the elbow joint from 100 to 135 degrees. Resistance during flexion and forearm supination with concurrent paresthesias suggests the LF may be a potential site of compression [20,41,57].

Middle finger proximal interphalangeal joint flexion against the examiner’s applied sustained resistance can be used to check the FDS as a site of possible compression [20,57]. However, this examination seems to be of low reliability because FDS contraction usually draws the lumbrical muscles into the carpal tunnel, which can compress the MN inside it, thus making it impossible to determine the exact location of compression required for diagnosis [12,84].

Adler et al. [12] state that numbness in the thenar eminence is a significantly more common symptom in patients with PS and is absent in CTS, because the palmar cutaneous branch of the MN originates proximal to the wrist crease and does not pass the carpal tunnel. Therefore, it remains unaffected by MN compression inside the carpal tunnel. In addition, CTS-specific tests—the Tinel, Phalen and carpal tunnel compression tests—are often negative in isolated PS [81,85,86].

The Hoffman–Tinel sign, commonly known as the Tinel sign, is defined as “pins and needles” induced by light percussion on a peripheral nerve which results in paresthesia in the distal cutaneous distribution of the nerve [86]. Nowadays, it is most associated with CTS and should be performed on the wrist [86]. The literature indicates that the Tinel sign can be present in the proximal forearm, but multiple studies have shown a positive result in under 50% of patients [11,19,48,83].

The Phalen test consists of complete flexion of the wrist for one minute without applying force. In patients with CTS, the flexed position of the wrist further compresses the MN and transmits a paresthesia to the region it innervates [81,87].

Carpal tunnel compression, proposed by Durkan [85], involves using both thumbs to apply direct pressure to the carpal region for 30 s. Rapid manifestation of CTS symptoms confirms a positive result [81]. All the tests that can be performed during the physical examination and help the clinician with diagnosis are summarized in Table 2.

There is also a hypothesis that muscle fatigue is more common in PS than in CTS, whereas thenar atrophy more often indicates CTS [4,88].

Although diagnosis should be based on symptoms and sensory alterations in the upper extremity, neurophysiological methods for measuring the conduction velocity of the MN can be useful when differentiating CTS from PS [87,89], as the latter is typically characterized by negative or inconclusive electromyographic and nerve conduction studies [12,88]. A positive result for PS is characterized by decreased conduction velocities from the elbow to the wrist [25], whereas a positive CTS result, the velocity is decreased at the mid-palm and past the carpal tunnel [90].

**Figure 1:**
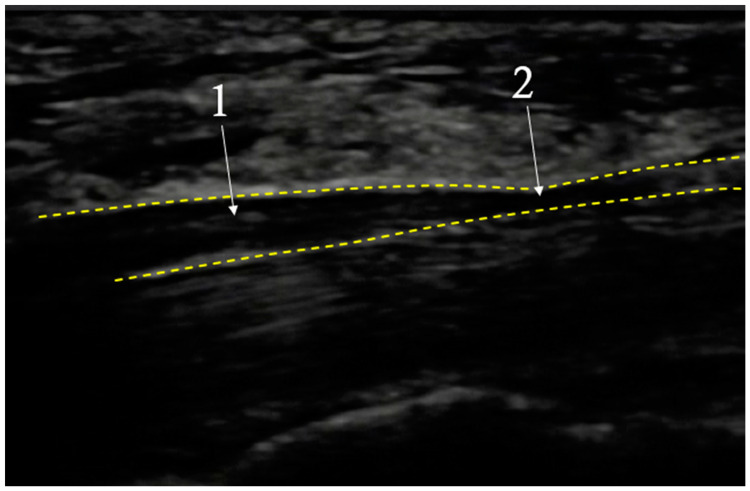
Exemplary image seen during USG examination of Carpal Tunnel Syndrome. CTS—dotted line; Medial Nerve (MN)—contours; 1—nerve dilatation before carpal tunnel; 2—nerve compression in carpal tunnel; longitudinal cross section.

In cases where there are symptoms consistent with CTS together with normal physical and electroneuromyographic examination results, high resolution ultrasonography (USG) can be useful for diagnosis, showing thickening of medial nerve distally to the compression site [91,92] (Mov. 1). Buchberger et al. [93] were the first to diagnose this syndrome using USG and their findings have been confirmed in previous magnetic resonance studies [94,95]. The current criteria used for detecting CTS in magnetic resonance and ultrasonography are: edema of the MN at the entrance to the carpal tunnel and flattening of the MN, and arching of the flexor retinaculum at its exit from the carpal canal [96]. The overall sensitivity of ultrasonography for CTS is lower than that of physical examination and electromyography [81]. A typical image seen during USG examination of CTS is shown in Figure 1.

**Figure 2:**
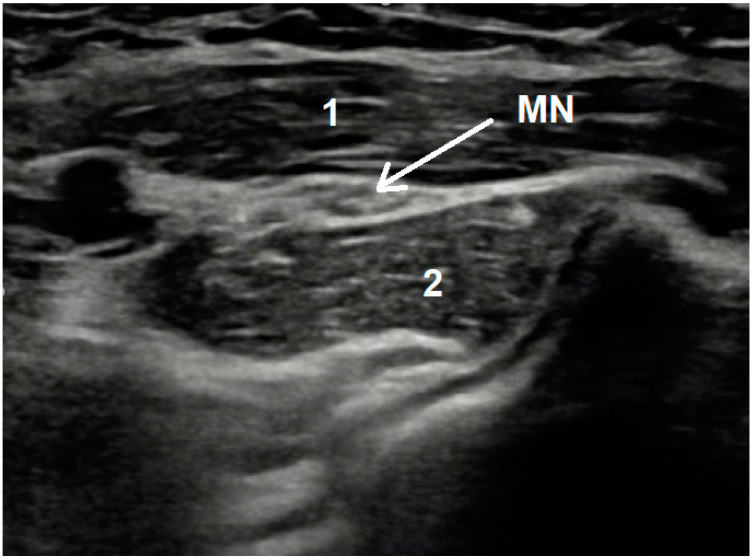
Exemplary image seen during USG examination of pronator syndrome showing medial nerve compression. MN—Medial Nerve; 1—humeral head of Pronator Teres muscle; 2—ulnar head of Pronator Teres muscle; longitudinal cross section Mov. 1 Forearm USG examination result showing median nerve thickening distally to compression site.

In contrast to CTS, both magnetic resonance and ultrasonographic imaging are not recommended in a PS workup because of the lack of current clinical evidence [12]. However, USG examination sometimes can visualize the MN compression site between PT heads—Figure 2.

During differential diagnosis, aside from CTS and PS, anterior interosseous nerve syndrome (AINS) should be considered owing to the course of this nerve near the FPL and MN and its possible compression in locations similar to those in CTS [44,97,98]. However, because the AIN is a purely motor nerve, patients should present only with muscle weakness and fatigue unless there is concurrent CTS [12]. EMG studies, which are often negative in PS, can confirm the diagnosis of AINS in 80% to 90% of patients, guiding the clinician to appropriate diagnosis of isolated AINS or AINS concomitant with CTS [98]. Moreover, Hagert and Hagert [19] emphasize that manual motor testing of pure MN- and AIN-innervated muscles can help to improve diagnostic accuracy.

## 7. Treatment of PS

Pronator syndrome treatment can be divided into conservative and surgical. All patients with diagnosed PS should be primarily managed with a nonsurgical approach because there is improvement in 29% to 80% of cases [24,37,88].

The most common choice is an initial period of three to six months of therapy, which involves nonsteroidal anti-inflammatory drugs and modification of activities that exacerbate symptoms (repetitive elbow flexion, forearm pronation and forceful gripping), local injections with corticosteroids or local anesthetics, rest, and activity modification [15,24,57,88,99]. Patients are allowed to work during therapy as long as there are no prominent motor or sensory deficits [100].

The data are too limited to support any specific duration or regimen for conservative treatment, so it remains challenging for clinicians to decide when surgical treatment should be proposed. However, surgery should be considered for any patient with persistent symptoms and an established diagnosis of PS if nonsurgical treatment has failed and there are one or more objective findings in a physical examination of weakness or motor atrophy, or abnormal electrodiagnostic results [12,99].

The main goal of surgery, regardless of the type of incision, is to release all suspected structures that can compress the MN [24,57]. The operational strategies in the available literature propose three main approaches:

1. Exploration of the MN in the forearm and release of the ligament of Struthers, PT, LF and fascia of the FDS in a single procedure [99,100]. This method was used owing to the limited reliability of prediction of compression sites along the course of the MN [24,57].
2. More limited decompression of the PS at only one or two most common sites of compression [11,19,48].
3. Endoscopic, mini-invasive release of specific compression sites [11,83]. One of the advantages of this technique is minimal interference with the nerve’s blood supply, but appropriate equipment and a qualified team are required [4].

Due to the fact that all surgical techniques are performed in the vicinity of the median nerve, there is a chance that it will be mechanically damaged during surgery. However, indirect damage can occur by damaging the nerve blood supply. Comparison of advantages, disadvantages, and potential complications for three main surgical treatment strategies are presented in Table 3.

## 8. Treatment of CTS

Like PS, CTS also can be managed conservatively or surgically. The decision is made by the evaluation of disease severity. In mild to moderate cases, conservative treatment is recommended [6,101]. Patients presenting with severe CTS symptoms or signs of nerve injury in electrodiagnostic examinations should be treated with surgery [6].

Non-operative therapy options include: splinting, corticosteroids, physical therapy, low-level laser therapy and therapeutic ultrasound [6,101,102] (Table 4). Maximal benefits can be observed after three months [103]. If there is no measurable improvement after six months, the clinician should offer surgical decompression [6].

Surgical treatment strategies for CTS can be divided into two major categories: open carpal tunnel release (OCTR) and endoscopic carpal tunnel release (ECTR) [117,118].

1. OCTR—well-established, generally accepted and relatively easier procedure in comparison to ECTR [119]. However, it is associated with potential complications such as persistent weakness, pillar pain, formation of hypertrophic scars in the incisions that cross the wrist, scar tenderness, slow recovery, and a higher incidence of persistent pain [120].
2. ECTR—established in response to complications encountered after OCTR. This procedure provides a faster recovery rate, allowing for smaller incisions; therefore, the esthetic effect is also better than in the case of OCTR [121]. Disadvantages of that strategy are mainly the special equipment and an operational team with appropriate qualifications and experience in this type of surgery [122]. Moreover, ECTR is more technically difficult, time-consuming, and associated with incomplete transverse carpal ligament (flexor retinaculum) release and neurovascular injury [122].

Surgical decompression provides a long-lasting positive outcome in 70% to 90% of cases [117]. Endoscopic and open surgery are equally effective; however, patients return to work on average eight days earlier after the endoscopic procedure, whereas the recovery time is usually longer after OCTR [6,118]. Some patients, especially those with severe CTS and treated with OCTR, need up to one year to experience full recovery [6,118]. Combining surgical treatment with splinting does not improve outcomes. Moreover, it can increase the formation of adhesions and stiffness [102].

## 9. Concurrent Carpal Tunnel and Pronator Syndromes

There is little information in the literature about carpal tunnel syndrome combined with PS, also called “double crush” [117,123,124]. Owing to its much higher prevalence in the general population, CTS is more frequently diagnosed [3,125]. However, if coexisting PS is overlooked, the symptoms will not be completely relieved after carpal tunnel release, forcing the patient to undergo further treatment and possibly surgery [123,124,126].

Typical symptoms are pain, tingling, and numbness in the radial three and a half digits [4]. According to Hsiao et al. [4], diagnosis of combined CTS with PS can be established on the basis of paresthesia involving the thenar eminence and proximal forearm. Additionally, nocturnal paresthesia symptoms are absent in PS. Therefore, if nocturnal symptoms occur in PS, CTS should also be considered [4]. The inclusion and exclusion criteria for the diagnostic system described by Hsiao et al. [4] are presented in Table 5, and this could be a valuable list for clinicians.

Owing to the low prevalence and the sparse supporting scientific data, no commonly accepted operating algorithms are available in the literature. However, the most commonly acknowledged treatment is invasive [4,117,123,124]. Hsiao et al. [4] performed arthroscopic release of the transverse carpal ligament and open decompression of the PT in patients undergoing surgery for the first time, with good outcomes: 71% showed complete relief of pain and paresthesia. The remaining 29% had occasional paresthesia and pain, but no sensory deficit.

Asheghan et al. [128] emphasize that addition of AIN conduction examination to standard clinical examination is a reliable way to differentiate between CTS, PS, or peripheral neuropathies; as the AIN is a purely motor nerve, patients experiencing its dysfunction should present only with muscle weakness and fatigue, unless there is concurrent CTS.

Luangjarmekorn et al. [129] compared the results of different treatments of suspected PS after failed primary CTS surgery. Their results indicate that patients with additional pronator teres release surgery show more chances of full recovery of numbness and pain than patients, in which only CTS surgery was done. Moreover, mean grip strength was also increased more in patients with additional pronator teres release.

Therefore:

− Every patient presenting a CTS should be carefully examined for the presence of PTS, due to overlapping symptoms or the possibility of coexisting pathology, that can lead to ineffective treatment strategy.
− If primary symptoms indicate PS, but the patient reports nocturnal paresthesia, coexisting CTS should also be considered.
− When suspecting coexisting CTS with PS, signs of paresthesia involving the thenar eminence and proximal forearm should guide the diagnosis towards its confirmation.
− Clinical diagnosis of PTS, in case of suspecting “double crush”, should be confirmed with ultrasonography or electrodiagnostic examination, especially when the invasive treatment strategy is considered. However, negative results should not rule out diagnosis of “double crush”.
− Probabilities of thoracic outlet compression syndrome, peripheral polyneuropathy, cervical radiculopathy, and cerebrovascular accident should be enrolled before proceeding with “double crush” diagnosis.
− Spine injuries, head injuries and upper limb injuries in medical history should increase the vigilance of the diagnosing team and should be excluded as a source of symptoms, before proceeding with “double crush” syndrome.
− Arthroscopic release of the transverse carpal ligament and open decompression of the PT in patients, in which concurrent CTS and PS is established as a diagnosis and are undergoing surgery for the first time, is a considerable technique, that is often met with satisfactory results.
− In patients with failed primary CTS surgery, in which PS was suspected, simultaneous release of the transverse carpal ligament and pronator muscle show more chances of full recovery of numbness and pain.

## 10. Summary

An appropriate theoretical background in anatomy and physiology is necessary to ensure effective treatment. Despite their many obscure aspects, carpal tunnel syndrome (CTS) and pronator syndrome (PS) are different entities and a properly conducted differentiation process should allow for appropriate diagnosis and treatment. Physical examination, the basis for diagnosis, should also consider the possible coexistence of CTS and PS. PS should be considered in the differential diagnosis for all patients with proximal forearm pain and MN paresthesias, whereas CTS should be considered for patients who report tingling, numbness and pain within the thumb and the index and middle fingers, worsening at night. Possible MN compression within the carpal tunnel is also indicated by thenar atrophy. Electrodiagnostic studies are often negative or unreliable in PS and often positive in CTS. The type of proposed treatment should be adjusted to the general condition of the patient and the severity of the symptoms. For mild to moderate symptoms in both syndromes, it is believed that treatment should be conservative. Surgical treatment should be administered in the event of severe symptoms or disturbing diagnostic test results, such as signs of MN injury.

Unfortunately, no commonly accepted algorithms have been designed for specific clinical circumstances; in addition, most current outcome data are based on retrospective case series with variable outcome measures by clinicians using their own surgical and physical examination techniques. There is hence a need for further and more structured research on this topic.

## Figures and Tables

**Table 1:** Causes of the chronic form of Carpal Tunnel Syndrome. *—defined as a distal radius fracture with dorsal comminution, dorsal angulation, dorsal displacement, radial shortening, and an associated fracture of the ulnar styloid [59].

Local Causes	Regional Causes	Systemic Causes
Inflammatory: Tenosynovitis Histoplasma fungal infection Hypertrophic synovium Trauma: Colles fracture * Carpal bone dislocation Tumors Lipoma Neuroma Ganglion Hemangioma Anatomical anomalies: Abnormal muscle bellies Bony abnormalities Thickened transverse carpal ligament	Osteoarthritis Rheumatoid arthritis Amyloidosis Gout	Diabetes Obesity Hypothyroidism Pregnancy Menopause Systemic lupus erythematosus Scleroderma Dermatomyositis Renal failure Long-term haemodialysis Acromegaly Multiple myeloma Sarcoidosis Leukemia Alcoholism Hemophilia

**Table 2:** Various tests performed during physical examination indicating possible sites of MN compression.

Test	Description	Possible Site of MN Compression (PS/CTS)
Pronator compression test	Applying sustained pressure for 30 s over PT in trajectory of MN. Paresthesias confirm positive test result.	Pronator teres muscle (PS)
Provocative forearm movements	Pronation of forearm against resistance with elbow joint flexed from 0 to 45 degrees. Presence of increased pain sensation or paresthesias confirm positive result of test.	Pronator teres muscle (PS)
	Flexion of elbow joint from 100 to 135 degrees. Resistance during flexion and forearm supination with concurrent paresthesias confirm positive test result.	Lacertus fibrosus (PS)
	Middle finger proximal interphalangeal joint flexion against applied sustained resistance. Paresthesias, pain or numbness confirm positive test result.	Flexor digitorum superficialis (PS) or carpal tunnel (CTS)
Tinel (Hoffman–Tinel) test	Light percussion on MN at wrist resulting in paresthesias experienced in distal cutaneous distribution of nerve confirm positive test result.	Carpal tunnel (CTS)
Phalen test	Complete flexion of the wrist for one minute without applying force. Paresthesias confirm positive test result.	Carpal tunnel (CTS)
Carpal tunnel compression (Durkan test)	Both thumbs directly applying pressure to the carpal region for 30 s. Rapid manifestation of CTS symptoms confirms positive result.	Carpal tunnel (CTS)

**Table 3:** Comparison of advantages, disadvantages, and potential complications for surgical treatment strategies.

Approach	Description	Advantages	Disadvantages	Complications	References
**Wide**	Release of the ligament of Struthers, Pronator Teres, Lacertus Fibrosus and fascia of the Flexor digitorum superficialis.	Provides the greatest likelihood of symptom relief in case of uncertainty about the compression site of the median nerve and provides better insight into the operational field.	May compromise nerve blood supply. This operating strategy can lead a relatively large scar formation.	Incomplete release of compressing structures, recurrent symptoms of pronator syndrome, nerve ischemia leading to motor or sensory deficits, longer recovery of muscle function.	[24,57,99,100]
**Limited**	Decompression of the one or two most likely nerve compression sites.	Limits the invasiveness of the procedure into the tissues providing quicker recovery of muscle function. This operating strategy leaves a smaller scar than the “wide” approach.	Greater risk of not releasing all compression sites of the median nerve. Requires greater precision in determining decompression sites. It may partially comprise nerve’s blood supply.	Incomplete release of compressing structures, recurrent symptoms of pronator syndrome therefore need to re-operate with wider approach.	[11,19,48]
**Endoscopic**	Mini-invasive release of specific compression sites.	Does not compromise the nerve’s blood supply, reduces scar formation, minimal invasiveness, minimal muscle function deficits and quick recovery.	Requirements for operational equipment, proper qualifications of the operational team. Requires greater precision in determining decompression sites. Limited view of the operational field.	Incomplete release of compressing structures, recurrent symptoms of pronator syndrome therefore need to re-operate with wider approach.	[4,11,83]

**Table 4:** Conservative treatment modalities for CTS.

Treatment Modalities	Description
Splinting	Most common conservative therapy for CTS [104] Neutral wrist position improves hemodynamic parameters, reducing the edema and minimizing nerve friction and compression [105] Less than 1% of patients discontinue treatment [106] Therapy lasts for three-month period at night-time [106] No evidence supporting full-time use of wrist splint rather than a night-only period [107] Recommended in pregnancy [102]
Local corticosteroid injection	Exact mechanism of the therapy remains unclear, but probably the anti-inflammatory factor is most significant in relieving symptoms [108] No objective standard for defining the ideal dose or specific drug [109] No significant clinical benefit was found for corticosteroid injections over other treatments, including splint immobilization [110] Possible adverse effects: infection, allergic reaction, osteonecrosis, tendon rupture and nerve or tendon injury [108] Temporary solution for patients with local or systemic risks for surgery [102]
Oral supplements and medications	Daily dose of vit. B6 proposed by authors is 200mg, but there are few research data to support this therapy [111] Adverse effects include numbness, paresthesia, and other symptoms related to sensory neuropathy [111] Oral steroids at low doses are more effective than nonsteroidal anti-inflammatory drugs and diuretics [112] Risk of side effects limits their long-term use [113]
Physical therapy	Gliding exercises can improve symptoms by stretching the adhesions among the tendons and the MN, decreasing tenosynovial edema, improving venous return and reducing pressure inside the carpal tunnel [114] Conflicting results of the effects of this therapy have been published [108]
Low-level laser therapy	Transfer of energy inducing increased production of endorphins, serotonin, and several mediators reducing the inflammatory reaction and increasing analgesia [115] Recommended dose is 8–10 J/cm^2^ with a wavelength ranging from 830–904 nm [115] Evidence of treatment efficacy still needs to be demonstrated and cost-effectiveness needs to be assessed [102,112]
Therapeutic ultrasound	Physical therapy that uses sound waves administered by a specific transducer and absorbed by surrounding tissue [116] Limited number of studies Poor quality evidence

**Table 5:** Inclusion and exclusion criteria for the Hsiao [4] “double crush” diagnostic system.

Inclusion Criteria	Exclusion Criteria
Clinical signs of CTS	Thoracic outlet compression syndrome [127]
Paresthesia over the palm region of MN innervation	Peripheral polyneuropathies
Forearm muscle pain and tenderness	Spine injury or head injury
Positive Phalen’s sign and PT provocation test	Stroke or other upper neuron disease
Positive Tinel’s sign at both the carpal tunnel and pronator teres muscle	Cervical radiculopathy
	Previous injuries to the affected limb
